# Inversion Algorithm of Fiber Bragg Grating for Nanofluid Flooding Monitoring

**DOI:** 10.3390/s20041014

**Published:** 2020-02-13

**Authors:** Noorhana Yahya, Chai Mui Nyuk, Ahmad Fauzi Ismail, Nazabat Hussain, Amir Rostami, Atef Ismail, Menaka Ganeson, Abdullah Musa Ali

**Affiliations:** 1 Department of Fundamental and Applied Science, Universiti Teknologi PETRONAS, Seri Iskandar 32610, Perak, Malaysia; mnchai88@gmail.com (C.M.N.); nazabat@gmail.com (N.H.); amir.phy@hotmail.com (A.R.); atef@usa.com (A.I.); menaka.ganeson@utp.edu.my (M.G.); alicorp07@gmail.com (A.M.A.); 2 Advanced Membrane Technology Research Centre (AMTEC), Universiti Teknologi Malaysia, Skudai 81310, Johor, Malaysia; afauzi@utm.my; 3 Department of Physics, Al-Azhar University, Assiut 71524, Egypt; 4 Geology Department, Bayero University Kano (BUK), Kano 700241, Nigeria

**Keywords:** nanotechnology, fiber Bragg grating, coupled-mode theory, inversion algorithm

## Abstract

In the current study, we developed an adaptive algorithm that can predict oil mobilization in a porous medium on the basis of optical data. Associated mechanisms based on tuning the electromagnetic response of magnetic and dielectric nanoparticles are also discussed. This technique is a promising method in rational magnetophoresis toward fluid mobility via fiber Bragg grating (FBG). The obtained wavelength shift due to Fe_3_O_4_ injection was 75% higher than that of dielectric materials. This use of FBG magneto-optic sensors could be a remarkable breakthrough for fluid-flow tracking in oil reservoirs. Our computational algorithm, based on piecewise linear polynomials, was evaluated with an analytical technique for homogeneous cases and achieved 99.45% accuracy. Theoretical values obtained via coupled-mode theory agreed with our FBG experiment data of at a level of 95.23% accuracy.

## 1. Introduction

Enhanced oil recovery is a tertiary method designed to recover the remaining oil left in a particular reservoir after primary and secondary methods are exhausted [1,2,3,4,5]. The nature and location of reservoir placement in the subsurface makes it difficult to estimate the amount of remaining oil and the position of mobilized oil in the reservoir.

The introduction of nanotechnology in enhanced oil recovery (EOR) has huge potential to increase total recovery in both light and heavy oil reservoirs. On the basis of previous studies [5,6,7,8,9,10,11], employing nanoparticles can shift reservoir wettability from oil-wet to water-wet and reduce oil viscosity. At high temperatures, however, nanoparticles can create a massive diffusion-driving force caused by a large surface-to-volume ratio [12,13,14], with the penetration of these tiny particles into pore spaces observable by using currently available technologies [15,16,17,18].

To address these problems, a new, advanced technology that can withstand a high-temperature high-pressure (HTHP) environments had to be be designed. Moreover, conventional methods are no longer applicable in high-temperature high-pressure environments. Low-frequency electromagnetic-wave energy can instead be used to stimulate oil in the reservoir due to its penetration depth. This method can enhance oil recovery via the interaction of nanomaterials in the form of nanofluids at the molecular level [19,20,21,22].

Magnetism and magnetic particles are widely used in many bioengineering and medical applications [23,24,25,26]. Magnetophoresis is a phenomenon in which force is exerted on a magnetic particle when it is subjected to a nonuniform magnetic field [27,28,29]. This phenomenon is potentially relevant to other applications, such as oil and gas, by remotely manipulating the properties of magnetic nanofluids.

This phenomenon can be further characterized as either positive or negative magnetophoresis [27]. Positive magnetophoresis is the migration of magnetic particles in a diamagnetic medium; negative magnetophoresis is the migration of diamagnetic particles in a magnetic medium. Magnetophoresis occurs in a magnetic-field gradient, a gradient of magnetization of the surrounding medium, or a combination of both. Magnetic permeability (l), flux density (B), and susceptibility (v) are the main elements to consider when designing and optimizing magnetophoresis applications.

Applying a magnetic field increases the viscosity of magnetic nanofluid, consequently improving fluid flow [30]. Magnetophoretic mobility not only contains information about the susceptibility of individual particles, but also reflects the particle size and viscosity of the fluent medium. The generated magnetic force is a function of magnetization saturation, particle volumes, and magnetic induction, as per below [31]:

(1) $$F_{mag}=\left(M_{s}V_{mag}\cdot \nabla \right)\vec{B}$$

where *F_mag_* is magnetic force, *M_s_* is magnetization saturation, *V_mag_* is particle volume, and $\vec{B}$ is magnetic induction.

On the basis of the literature, Yang et al. [32] reported on the direct coupling of a magnetic field with an electromagnetic (EM) wave in a Bragg sensor using a TbFeCo thin film (84–285 nm) as cladding; nevertheless, the magnetostrictive contribution could not discern the true influence of magneto-optic effects [33]. In addition, Pu et al. [34] developed ferrofluid as fiberoptic cladding to alter light transmission in a general single-mode fiber, but the ferrofluid’s magnetic response was slow (i.e., Hz) and dictated by particle motion in the fluid [35] rather than ferromagnetic resonance (GHz). Moreover, the transmission-based magneto-optic coupling was not conducive to multiplexing many sensors onto a single fiber.

The Faraday effect is a well-documented example of a magneto-optic effect that alters the imaginary component of permittivity, but without the effective index changes associated with real polarization directions. Therefore, further research is necessary to develop and understand the response of fiberoptic sensors integrated with magnetic sensing capabilities.

The Bragg wavelength of optical fiber grating is a function of the grating period (Λ) and the effective refractive index (*η_eff_*) of the fiber core, represented by Equation (2).

(2) $$\lambda_{B}=2\eta_{eff}\Lambda$$

Magnetic fluid is an example of a stable colloidal solution composed of ferromagnetic nanoparticles. The behavior of ferromagnetic particles that appear in magnetic fluids is dependent on the external magnetic field, so the refractive index of magnetic fluid can be seen to be magnetic-field-dependent [36,37,38]. Refractive index *η* is as below [36]:

(3) $$\eta =\sqrt{\mu_{r}},$$

where $\mu_{r}$ represents relative permeability.

Nuclear magnetic resonance (NMR) is used for the real-time quantitative detection of multiphase flow in oil and gas wells and pipelines (Shi et al., 2019). The advantages of NMR include noninvasive flow detection, environmental protection, and full oil, gas, and water three-phase range. The considered fiber Bragg grating (FBG) detector, however, boasts an interaction with these nanofluids that was not reported elsewhere. The novelty of the FBG sensor is on its application in nanofluid-enhanced oil recovery (EOR) where it is used to monitor the flow of mobilized fluid in a reservoir.

In existing core-flooding systems, the delineation of fluid mobilization and magnetic-field strength have not been developed. Oil mobilization can instead be detected using optical sensors followed by a computer algorithm to convert these sensor data and predict an image of oil movement [39].

The main objective of our research was to develop an adaptive computational algorithm based on finite difference and coupled-mode theory to image out oil mobility inside a porous medium. The following sections summarize the main ideas behind the involved optical sensors, magnetization, coupled-mode theory, and computational algorithms.

## 2. Methodology

### 2.1. Molecular-Dynamics Simulation

In this work, an Angsi oilfield sandstone structure with 24% porosity, butane as oil, and 10,000 ppm of brine (H_2_O + NaCl) was simulated and optimized by the Forcite module of software suite Materials Studio 18.1. Nanoparticle structures were imported from the library of materials inside this software. Van der Waals interactions between different particles were calculated within the framework of the Lennard-Jones (LJ) potential. Molecular-dynamics (MD) simulations were completed in the canonical ensemble (NVT—amount of substance (N), volume (V), and temperature (T)) with a time step of 1 fs. A Nose thermostat was used to keep the temperature at 343.15 K, and a universal force field was then applied via an Ewald electrostatic method.

In the MD simulation, the stress autocorrelation function is a summative function that can be used for estimating pressure correlation between two surfaces. Therefore, it takes into account the total effects of all atoms involved in the process [40], expressed as

(4) $$C_{xy}\left(t\right)=\langle \sum_{x<y}P_{xy}\left(t\right)P_{xy}\left(0\right)\rangle ,$$

where $P_{xy}$ refers to an independent component of stress in the *xy* direction (or shear stress).

For molecular fluid, two formalisms can be employed to estimate the stress tensor: atomic and molecular formalisms with minimal variance in the obtained result [41]. The stress tensor can be calculated on the basis of the motion of individual atoms in the system, as per below:

(5) $$P^{\left(a\right)}\;V\;=\;\sum_{i,a}m_{ia}v_{ia}v_{ia}\;+\;\sum_{i,a}r_{ia}f_{ia},$$

where $m_{ia,}r_{ia}$, $v_{ia}$, and $f_{ia}$ are mass, position, velocity, and force on an atom of molecule I, respectively. For molecular formalism, stress-tensor calculation is based on molecule motion in the system, given as:

(6) $$P^{\left(m\right)}\;V\;=\;\sum_{i}m_{i}v_{i}v_{i}\;+\;\sum_{i}r_{i}f_{i},$$

where $m_{i,}r_{i},v_{i}$ and $f_{i}$ are mass, center of mass position, center of mass velocity, and the total force on molecules, respectively. Thus, a stress-autocorrelation function (SACF) can be employed to estimate shear stress between oil and rock surface, with and without nanoparticles.

### 2.2. Experiment Work

Our model comprised a cylindrical container as the core, glass beads as sandstone, and an iron rod with 60 turns of copper wire (solenoid) as a magnetic transmitter connected to an electromagnetic machine with a frequency of 200 kHz and FBG. The container was filled with glass beads representing sandstone in a porous medium and initially filled with 20% oil and 80% brine. Five experiments with 50 mL of brine-based nanofluid containing different nanoparticles (NPs), namely, hematite (Fe_2_O_3_), magnetite (Fe_3_O_4_), zinc oxide (ZnO), aluminum oxide (Al_2_O_3_), and carbon nanostructure (CNS), was then injected in the presence of a 200 kHz EM field. Wavenumber counts were recorded.

### 2.3. Simulation Setup

**Figure 1:**
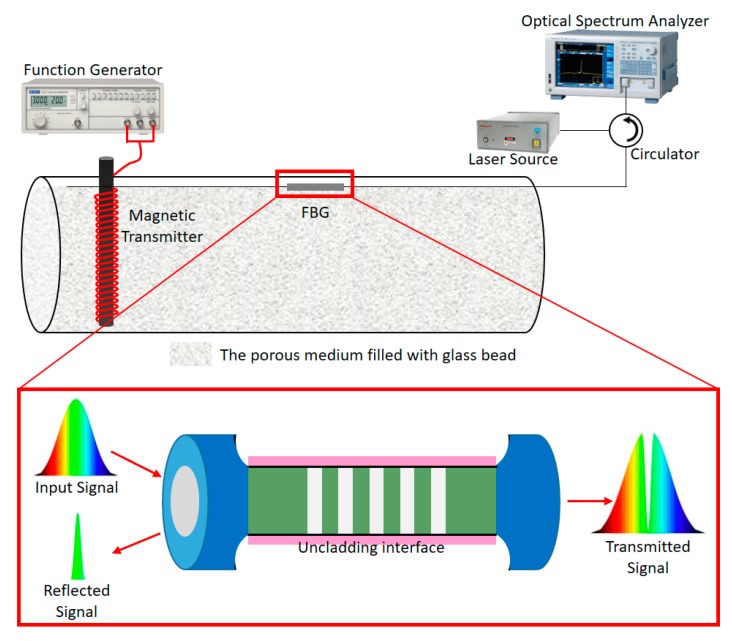
Schematic of fiber Bragg grating (FBG) experiment.

The equivalent form of a two-dimensional solution domain of core flooding was truncated, as seen in in Figure 1. The length and height of the solution domain were 30.90 and 3.80 cm, respectively. The domain of interest was discretized into 46,968 cells, and the dimensions of each cell were 500 × 500 µm. Both etched FBG sensors (FBG 1 and 2) were used to sense the strength of the magnetic field radiated by a solenoid source. FBG 1 and 2 were used to investigate the spectral reflectivity, bandwidth, and side lobes at Bragg wavelengths of 1534 and 1552 nm, respectively. The physical orientation of FBG 1 and 2 was assumed to be 20.0 and 30.0 cm, respectively, in the direction of the *x*-axis.

**Figure 2:**
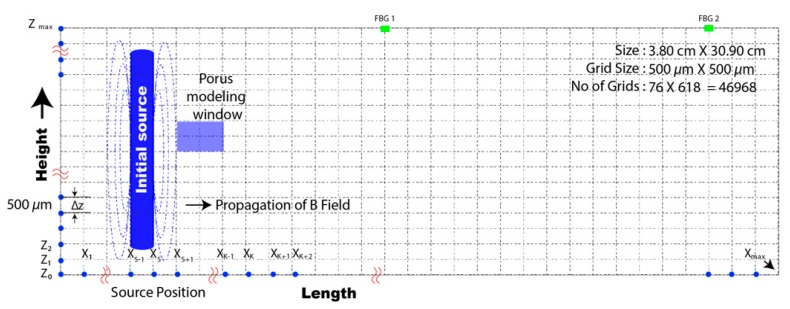
Two-dimensional domain of interest with solenoid-based magnetic source.

Strength and diffusion rate were determined by iteratively applying a computational algorithm to solve the governing field equation of our magnetic field. Source modeling for the aforementioned simulation setup was then implemented in COMSOL Multiphysics software, and the source beam was inserted into the proposed computational algorithm. The 2D domain of interest with our solenoid-based magnetic source is shown in Figure 2.

The intensity of the time-varying magnetic field was sensed by the FBG sensor in our real-time environment. Variation in intensity and the Bragg shift of sensed optical data occurred because of changes in the magnetic field and fluid dynamics. The strength of the magnetic field in our region of interest was computed by iteratively solving Equation (7):

(7) $$\nabla^{2}H-i\mu \omega \sigma H=0$$

The magnetization force was given as

(8) $$M=\chi_{V\left(magnetite\right)}H,$$

where $\chi_{V\left(magnetite\right)}$ was the volume magnetic susceptibility for magnetite.

This magnitude of magnetization force was used to calculate the value of the refractive index as below:

(9) $$\eta^{2}=aM+b,$$

where *a* and *b* were proportional constants, and M was magnetization.

This explains that the magnetization of our ferromagnetic materials was caused by the exposure of coated magnetite nanoparticles to an external magnetic field. The refractive index of magnetite nanoparticles changed following the fluctuation of magnetization at different external magnetic fields. A stronger magnetic field led to a higher refractive index.

**Figure 3:**
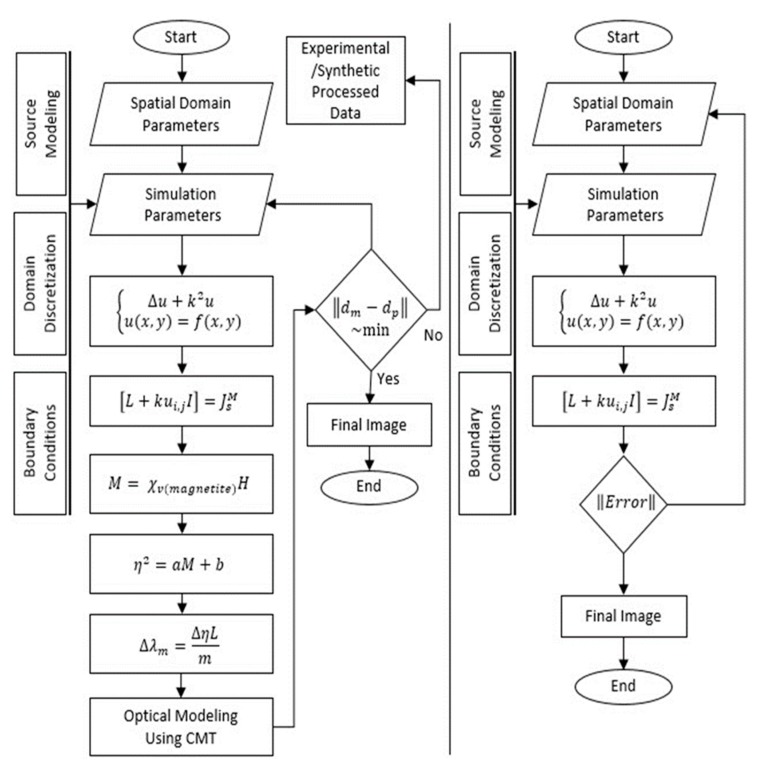
Flow diagram for inversion of optical data using iterative algorithm.

**Figure 4:**
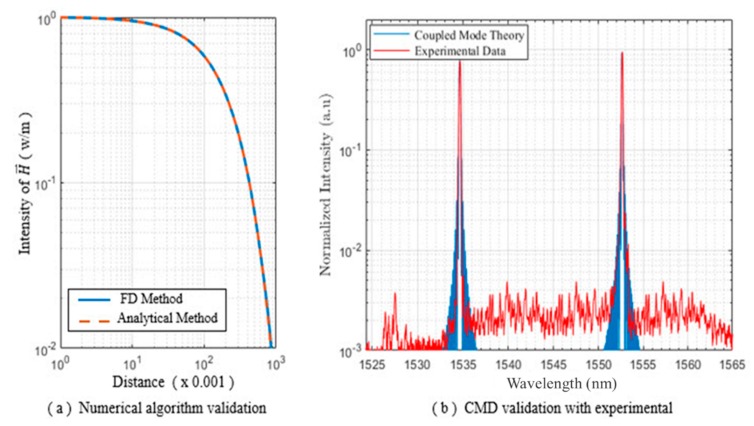
Numerical validation of proposed algorithms based on analytical solution and experiment data.

**Figure 5:**
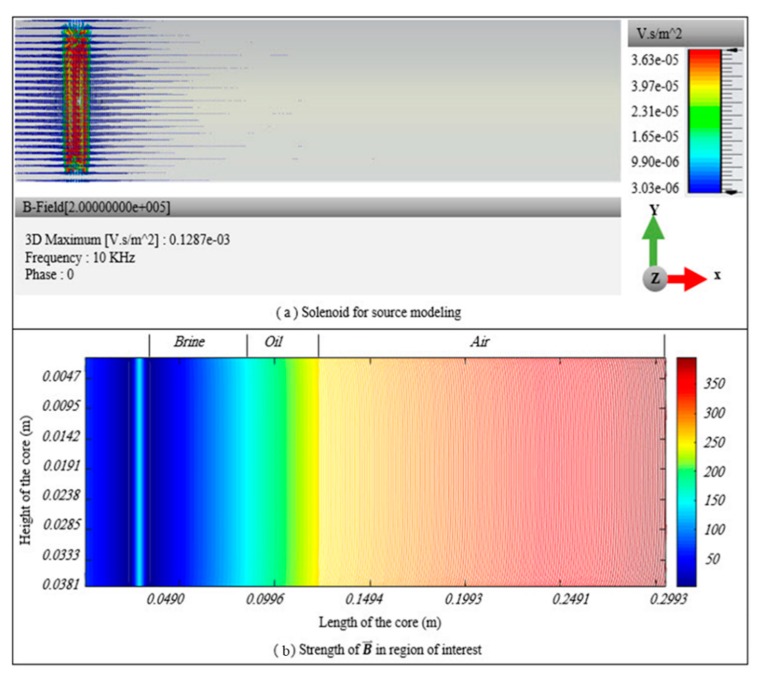
Source modeling and magnetic-field profile during fluid motion in porous medium.

This process was iteratively performed to reduce the distance between modeling data (*d_m_*) and processed data (*d_p_*). Figure 3 shows the complete flow diagram of the adaptive iterative algorithm that predicted our profile of oil mobilization in a region of interest. Figure 4 shows the numerical validation of the proposed algorithms based on our analytical solution and experiment data. Finally, source modeling and a magnetic-field profile during fluid motion in the porous medium is shown in Figure 5.

## 3. Results

### 3.1. Simulations of Fe_2_O_3_, Fe_3_O_4_, ZnO, Al_2_O_3_, and CNS

**Figure 6:**
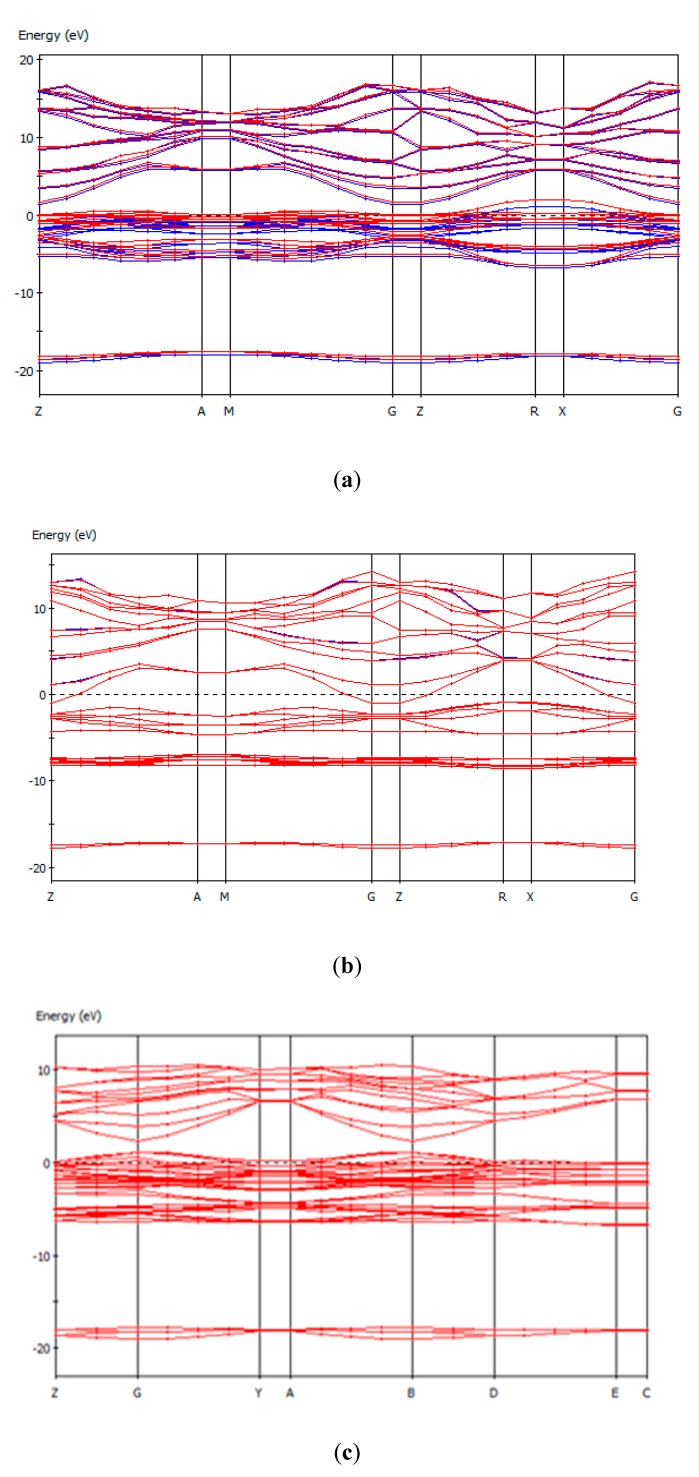

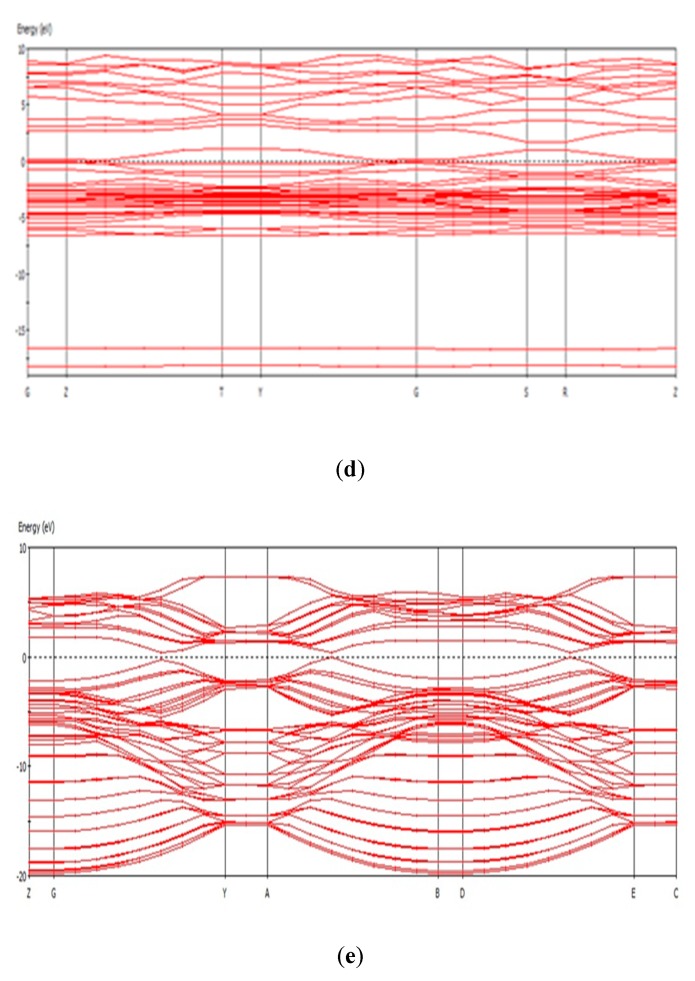
Band structure of (**a**) Fe_2_O_3_, (**b**) Fe_3_O_4_, (**c**) ZnO, (**d**) Al_2_O_3_, and (**e**) CNS.

In solid-state physics, band structure, otherwise known as the electronic band structure of a solid, describes the range of energies that an electron within a solid may or may not have. These existing energy bands are the allowed bands, while bands that do not contain energy are called energy gaps or forbidden bands. Band theory is used to describe physical properties, such as electrical resistivity and optical absorption. Figure 6 shows the band structure of (a) Fe_2_O_3_, (b) Fe_3_O_4_, (c) ZnO, (d) Al_2_O_3_, and (e) CNS.

### 3.2. FBG Response for Fe_2_O_3_, Fe_3_O_4_, ZnO, Al_2_O_3_, and CNS

**Figure 7:**
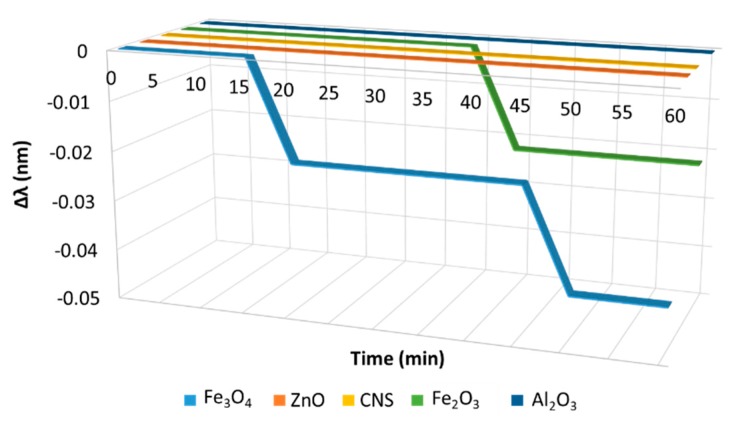
Wavelength changes over time during Fe_3_O_4_, Fe_2_O_3_, ZnO, Al_2_O_3_, and CNS injection.

Our research was designed to investigate the effects of FBG through the use of various fluid-based NPs for magnetic transmission at a frequency of 200 kHz. In this manner, we determined the most effective nanofluids that reacted with the FBG. Figure 7 illustrates the graph of FBG wavelength shift versus time as Fe_2_O_3_, Fe_3_O_4_, ZnO, Al_2_O_3_, and CNS were injected.

### 3.3. Numerical Algorithm Based on Finite-Difference Technique

**Figure 8:**
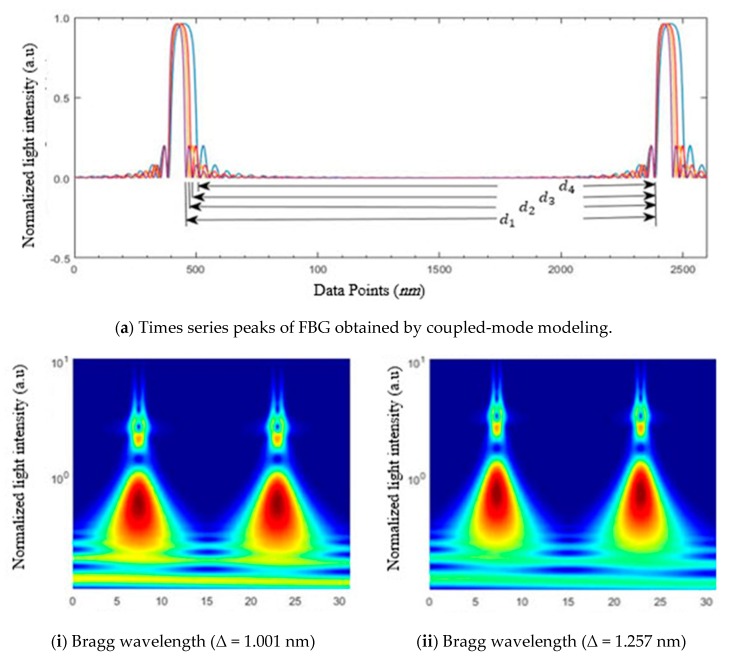

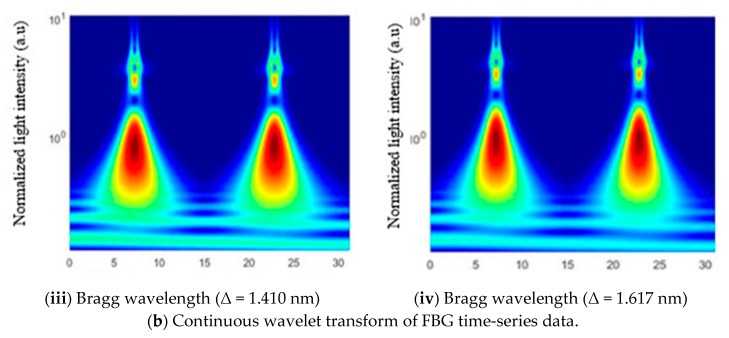
Theoretical optical-sensor time-series data obtained by coupled-mode modeling.

In this research, a numerical algorithm based on a finite-difference (FD) technique was used and validated via the aforementioned analytical solution. Obtained results for both the analytical and the adaptive numerical algorithm are shown in Figure 8a; the magnetic-field intensity curves exactly matched our proposed analytical solution. The relative error of the proposed algorithm was found to be 0:005429 for N_x_ = 1000 data points.

**Figure 9:**
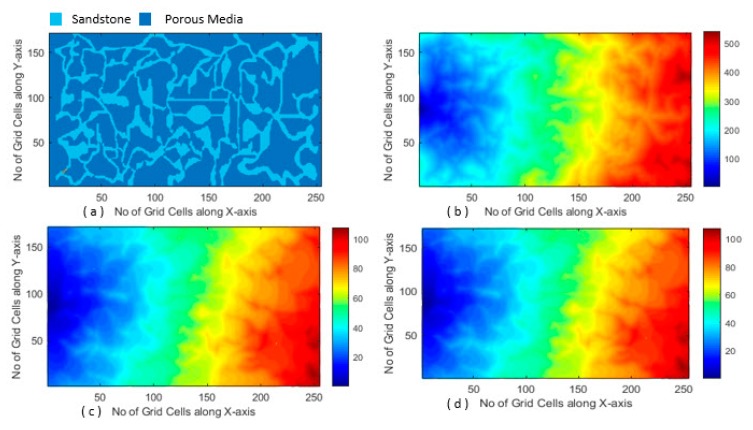
(**a**) Structure of porous medium and magnetic-field distribution in porous medium filled with (**b**) air, (**c**) oil, and (**d**) brine.

Prior to implementation of our proposed algorithm, source modeling was performed in CST EM Studio (CST2012 version), and the obtained results are shown in Figure 8b. Figure 9 shows the magnetic-field distribution in a porous medium. The intensity of the magnetic field inside our core container was very weak.

## 4. Discussion

Dielectric materials have a large band gap due to being electrical insulators, exhibiting more energy excitement from valence to covalent bands and allowing electrons to move freely and conduct electricity (Figure 6). The formation of these bands is mostly a feature of the outermost electrons in an atom [42]. Band gaps (Table 1) are essentially leftover energy ranges that are not covered by any band and that resulted from finite widths of energy bands, with widths dependent upon the degree of overlap in the atomic orbitals from which they arise [43].

It is clear from Figure 6 that the upper half of the electronic band structure (above Fermi level) described the π*- energy-antibonding band and the lower half (below the Fermi level) described the π energy-bonding band. Fermi energy lies exactly at the Dirac point: the π band was completely filled, while the π* band was empty; π* and π bands both degenerate at the K-point (Dirac point). Energy dispersions gradually deviated from linear relation in the higher-energy region [44]. As seen in Figure 7, the overall trend of the FBG response showed that there was a wavelength shift during Fe₃O₄ injection. No wavelength shifts occurred for ZnO, Al_2_O_3_, and CNS over that time. This is due to the activation of magnetic material (Fe_3_O_4_) by our magnetic field.

On the basis of EM theory, the propagation of a magnetic field happens in a horizontal vector that gives a plane wave to the horizontal FBG. Magnetic fluid is a kind of stable colloidal solution of ferromagnetic nanoparticles. The behavior of ferromagnetic particles in a magnetic fluid is dependent on the external magnetic field, so the refractive index of magnetic fluid tends to be magnetic-field-dependent. The wavelength shift of FBG was due to magnetic force *F_m_* that is represented by the following equation [45]:

(10) $$∆\lambda_{Bragg}=\left(1+p_{e}\right)\lambda_{Bragg}\frac{∆F_{m}}{EA},$$

where $p_{e}$ is the effective strain-optic coefficient, *E* is Young’s modulus, and *A* is the cross-sectional area. Thus, the recovery factor of our injected nanofluid Fe_3_O_4_ in the presence of a magnetic field and at a frequency of 200 kHz was 15% higher than that without EM application, as seen in Table 2. The FBG response for Fe_3_O_4_ was 75% higher than that for Fe_2_O_3_ due to the activation of Fe_3_O_4_ through EM application.

We used a magnetic transmitter so that only magnetic material would have a wavelength-shift response of FBG towards the nanofluid’s injection. Fe_3_O_4_ nanofluid material was chosen to validate our adaptive algorithm. The exponential form of the analytical method used to predict our electromagnetic field was also used for validation.

The gradient of magnetic-field intensity shown in Figure 8 shows that the maximum density field remained inside the solenoid, while the outer side of magnetic field intensity was sybaritically weak. Figure 5a shows the maximum value of the magnetic field inside the solenoid started at 3.63 × 10^−5^ V.s/m^2^, and blue lines indicated that the value of magnetic-field intensity within a few centimeters dropped to 3.03 × 10^−6^ V.s/m^2^. The source waveform for the adaptive algorithm was obtained by using data from a modeled source in COMSOL Multiphysics software.

The proposed algorithm was used to iteratively solve a governing field equation for each node of the discretized domain. The source waveform was also introduced exactly at the same location as that discussed in Figure 1. The electrical properties of the porous medium were activated by filling up 24% of the pores with brine, oil, or air. Inside our solenoid, perfect conducting properties were introduced according to our lab’s experiment setup, and the transmitter source was allowed to radiate magnetic flux towards the surrounding medium.

In order to plot our results, an arbitrary unit was introduced to our plot contour, as can be seen in the graphed gradient of Figure 9. The diffusion of magnetic field in brine-, oil-, and air-filled pores can clearly be seen. Deriving from Maxwell’s equations, the attenuation factor of the propagated EM wave can be generally written as [46]:

(11) $$\alpha =\omega \sqrt{\frac{\mu \varepsilon }{2}\left[\sqrt{1+{\left(\frac{\sigma }{\omega \varepsilon }\right)}^{2}}-1\right]},$$

where σ, ε, and μ represent electric conductivity, permittivity, and the magnetic permeability of the medium, respectively, and ω is the angular frequency of the emitted EM wave. For our air-filled porous medium, conductivity was negligible, and the attenuation factor in Equation (11) approached zero. However, for oil- and brine-filled pores (σ/ωε)≫1 we thus obtained:

(12) $$\alpha =\sqrt{\frac{\mu \sigma \omega }{2}}.$$

Equation (12) indicates low-frequency EM wave propagation. The strength of the magnetic field in the air-filled pores, with a negligible attenuation factor, was dramatically higher than that in the oil- and brine-filled pores, which indicated a more significant attenuation factor. In comparison with the low-conductive oil-filled pores, magnetic-field strength dropped off quickly in the conductive brine-filled pores.

Magnetic nanoparticles mixed with brine/oil were injected into the pores, and each of these particles experienced Lorentz force. The timed-varied strengths of the magnetic field caused the motion of these charges. The diffused magnetic field and magnetization force changed the cladding refractive index of our FBG sensor [47]. The intensity and Bragg wavelength shift of two consecutive FBG peaks are shown in Figure 8a. The distance between fall time and rise time for each main peak are indicated by d1–d4. In this case, d1–d4 indicate refractive-index values of 1:001, 1:257, 1:410, and 1:617, respectively.

Continuous wavelet transformation was used to investigate temporal changes in the optical data of two consecutive peaks, as illustrated for each case in Figure 8b. The FBG response was a narrow-band reflective filter that was centered at the Bragg wavelength, with the spectral response for uniform FBG affected by grating length and refractivity. Spectral reflectivity was a summation of $\eta_{eff}^{\infty }$ and $\eta_{eff}^{cl}$, in which $\eta_{eff}^{co}$ was independent of the surrounding environment and constant. However, $\eta_{eff}^{cl}$ was dependent on magnetization-force strength sensed by our FBG sensors.

## 5. Conclusions

Wavelength shift only occurred for a horizontal FBG setup during Fe_3_O_4_ injection. This indicates that the magnetic material (Fe_3_O_4_) was activated by EM waves. This paper showcases an adaptive algorithm to simulate oil mobilization in the presence of a magnetic field and the resultant responses sensed by etched optical sensors based on FBG. An equivalent theoretical model based on an adaptive algorithm was then implemented. The considered algorithm was validated by comparing existing analytical solutions with a respective error of 0.005429. Our study clearly shows the ability to compute an adaptive algorithm to predict oil mobilization. We found that the proposed computational method displays robustness with respect to the perturbation parameter in approximating a solution.

## Figures and Tables

**Table 1:** Electronic nanomaterial parameters.

Nanoparticle	Fe_2_O_3_	Fe_3_O_4_	ZnO	Al_2_O_3_	CNS
Number of electrons	28	204	36	144	112
Net system charge	0	0	0	0	0
Number of upspins	18	21	0	0	0
Number of downspins	10	21	18	72	56
Net system spins	8	0	18	72	56
Numbers of bands	22	159	22	87	68
Band gap (eV)	0.021	0.016	1.678	2.829	0.389
Stress-autocorrelation function (SACF)	−0.011	−0.019	7.597 × 10^−3^	1.339 × 10^−3^	1.931 × 10^−4^

**Table 2:** Wavelength shift and recovery factor of injected Fe_3_O_4_ nanomaterials.

Parameter	Wavelength Shift (nm)	Recovery Factor (%)
0 Hz	0.01	55
200 kHz	0.04	64
